# Association between vitamin b_12_ levels and melancholic depressive symptoms: a Finnish population-based study

**DOI:** 10.1186/1471-244X-13-145

**Published:** 2013-05-24

**Authors:** Jussi Seppälä, Hannu Koponen, Hannu Kautiainen, Johan G Eriksson, Olli Kampman, Jaana Leiviskä, Satu Männistö, Pekka Mäntyselkä, Heikki Oksa, Yrjö Ovaskainen, Merja Viikki, Mauno Vanhala, Jussi Seppälä

**Affiliations:** 1 Department of Psychiatry, South-Savo Hospital District, Mikkeli, Finland; 2 Department of Psychiatry, Institute of Clinical Medicine, University of Eastern Finland, P.O. Box 1627, Kuopio, 70211, Finland; 3 Department of Psychiatry, Kuopio University Hospital, P.O. Box 1777, Kuopio, 70211, Finland; 4 Unit of Family Practice, Central Finland Central Hospital, Jyväskylä, 40620, Finland; 5 Unit of Primary Health Care, Kuopio University Hospital, P.O. Box 1777, Kuopio, 70211, Finland; 6 Department of General Practice and Primary Health Care, University of Helsinki, P.O. Box 33, Helsinki, 00014, Finland; 7 Unit of General Practice, Helsinki University General Hospital, P.O. Box 590, Helsinki, 00029, Finland; 8 National Institute for Health and Welfare, P.O. Box 30, Helsinki, 00271, Finland; 9 Folkhälsan Research Center, P.O. Box 63, Helsinki, 00014, Finland; 10 Vasa Central Hospital, Vasa, 65130, Finland; 11 Medical School, University of Tampere, Tampere, 33014, Finland; 12 Department of Psychiatry, Seinäjoki Hospital District, Seinäjoki, 60220, Finland; 13 Department of Chronic Disease Prevention, National Institute for Health and Welfare, P.O. Box 30, Helsinki, 00271, Finland; 14 Institute of Clinical Medicine, General Practice, University of Turku, Turku, 20014, Finland; 15 Unit of Primary Health Care, Turku University Hospital, Turku, 20520, Finland; 16 Tampere University Hospital, P.O. Box 2000, Tampere, 33521, Finland; 17 Private practices, Diacor, Helsinki, 00380, Finland; 18 Medical School, University of Tampere, Tampere, 33014, Finland; 19 Tampere Mental Health Center, P.O. Box 487, Tampere, 33101, Finland; 20 Department of Psychiatry, South-Savo Hospital District, Moisiontie 10, Mikkeli, FIN, 50520, Finland

**Keywords:** Beck depression inventory, Melancholic depressive symptoms, Non-melancholic depressive symptoms, Population-based, Vitamin B_12_

## Abstract

**Background:**

An association between vitamin B_12_ levels and depressive symptoms (DS) has been reported in several epidemiological studies. The purpose of this study was to evaluate vitamin B_12_ levels in population-based samples with melancholic or non-melancholic DS as the relationship between vitamin B_12_ levels and different subtypes of DS has not been evaluated in previous studies.

**Methods:**

Subjects without previously known type 2 diabetes, aged 45–74 years were randomly selected from the National Population Register as a part of the Finnish diabetes prevention programme (FIN-D2D). The study population (N = 2806, participation rate 62%) consisted of 1328 men and 1478 women. The health examinations were carried out between October and December 2007 according to the WHO MONICA protocol. The assessment of DS was based on the Beck Depression Inventory (BDI, cut-off ≥10 points). A DSM-IV- criteria based summary score of melancholic items in the BDI was used in dividing the participants with DS (N = 429) into melancholic (N = 138) and non-melancholic DS (N = 291) subgroups. In the statistical analysis we used chi-squared test, *t*-test, permutation test, analysis of covariance, multivariate logistic regression analysis and multinomial regression model.

**Results:**

The mean vitamin B_12_ level was 331±176 pmol/L in those without DS while the subjects with non-melancholic DS had a mean vitamin B_12_ level of 324 ± 135 pmol/L, and those with melancholic DS had the lowest mean vitamin B_12_ level of 292±112 pmol/L (p < 0.001 after adjusted for age, sex, use of antidepressive medication and chronic diseases sum index). The adjusted difference of vitamin B_12_ levels between the non-melancholic and the melancholic group was 33 pmol/L (95%CI 8 to 57, p = 0.008). Melancholic DS and vitamin B_12_ levels showed an independent linearly inverse association. The relative risk ratio (RRR) for melancholic DS was 2.75 (95%CI 1.66 to 4.56) in the lowest vitamin B_12_ level tertile versus the highest (p for linearity <0.001) when those without DS formed the reference group. The RRR in the non-melancholic subgroup was nonsignificant.

**Conclusions:**

The vitamin B_12_ level was associated with melancholic DS but not with non-melancholic DS.

## Background

Depression is a global public health problem particularly in developed countries. Recently, the World Health Organization estimated that unipolar depressive disorder remains one of the leading causes of total disability adjusted life years (DALY’s ) worldwide [1]. It accounts for 8% of total DALY’s in the Americas and 6% in Europe [2]. Overall, the 12-month and lifetime prevalence rates of depression are approximately 12% and 24% among U.S. men and women, respectively [3].

A wide array of etiological hypotheses has been suggested to underlie depression. Of the biological hypotheses, the monoamine hypothesis proposes an important etiological role for serotoninergic or noradrenergic dysfunction in depression [4]. Besides folate, vitamin B_12_ is involved in single-carbon transfer reactions needed for the production of serotonin and other monoamine neurotransmitters [5]. Vitamin B_12_ deficiency may also result in the accumulation of homocysteine, which has been suggested to lead to exito-toxic reactions and may enhance depression [6,7]. Homocysteine can be remethylated to methionine, which requires vitamin B_12_[8]. Methionine is the immediate precursor of S-adenosylmethionine (SAM), the methyl donor of numerous methylation reactions in the brain, many of which are directly involved in the synthesis and metabolism of dopamine, norepinephrine and serotonin [9]. These findings form a plausible link between vitamin B_12_ and mood, and may also indicate that the association between depression and vitamin B_12_ could be mediated through monoamine synthesis.

In clinical studies, lower vitamin B_12_ levels have been found to be associated with severe depression [10,11]. Low serum vitamin B_12_ levels are also detected in approximately 20% of psychiatric patients [12]. On the other hand, high vitamin B_12_ levels were associated with a good treatment outcome in patients with major depressive disorders in a clinical setting [13]. However, a small randomised trial found no improvement in depression after the administration of vitamin B_12_ as an adjuvant [14].

The three cross-sectional studies and one prospective population-based study that have reported a connection between vitamin B_12_ levels and DS or depressive disorders were conducted on older adults [15-18]. In the Women’s Health and Aging Study B_12_ deficiency was associated with a two-fold increased risk of severe depression [15]. The Rotterdam Study found that B_12_ deficiency was independently associated with depressive disorder among older adults [16]. A study among Chinese older adults also found a correlation between deficient levels of vitamin B_12_ and greater risk of DS [18]. The only existing community-based prospective study reported that lower levels of vitamin B_12_ at baseline were associated with a higher risk of incident of depression on 2–3 year follow-up among older Korean people [17]. However, previous results have been somewhat inconsistent, since some studies, mainly conducted in younger populations, have found no association between vitamin B_12_ levels and DS or depressive disorders [19-23].

## Methods

### Study population and the setting of the study

The Finnish type 2 diabetes (FIN-D2D) survey is the implementation project of a national programme for the prevention of type 2 diabetes covering a population of 1.5 million during the years 2003–2008 [24]. The specific aims were to improve the screening of people at risk of diabetes and the detection of undiagnosed diabetes, as well as the prevention of diabetes among national population.

A random sample of 4500 subjects without previously known type 2 diabetes, aged 45–74 years, stratified according to gender and 10-year age groups (45–54, 55–64 and 65–74 years), was selected from the National Population Register of Finland in August 2007. The sampling represented three separate geographical areas with both urban and rural populations. The study participants were invited by mail to a health examination. The study population (N = 2806, participation rate 62%) consisted of 1328 men and 1478 women. The participants and the nonparticipants (N = 1694) did not differ with regard to age or gender distribution. The Ethical Committee of the Hospital District of Helsinki and Uusimaa approved the study protocol. All participants gave their written informed consent prior to participation in the study.

### Design of the study and measurements

#### Depressive symptoms

DS were assessed by using the Beck Depression Inventory (BDI), which is a 21-item self-report questionnaire consisting of symptoms and attitudes related to depression [25]. The items are summed in a total score with a range from 0 to 63. The cut-off point for DS was 10, which has been reported to be a feasible instrument for depression screening [26]. It has also been shown to be a useful tool for detecting depressive symptoms in various adult populations [27-32]. Out of the whole study population, 429 (15%) subjects with a BDI score ≥10 were identified. In order to examine the effect of the subtype of DS, we used a summary score of melancholic symptoms in the BDI based on the DSM-IV- defined criteria for melancholic depression (sadness, past failure, loss of pleasure, guilty feelings, punishment feelings, loss of interest, irritability, change of sleeping and appetite) in dividing the participants with increased DS into melancholic and non-melancholic depressive symptom subgroups in a similar way that has been published to be useful in several previous studies. The subjects were defined to have DS with melancholic characteristics when the number of melancholic symptoms exceeded the number of non-melancholic symptoms [29,33-36]. When using this method the subject had to score 2 or 3 points in each chosen item in order to have a positive item (a melancholic or a non-melancholic item).

#### Laboratory analysis

The study methods followed the World Health Organization MONICA protocol [37]. After an overnight fast, blood samples were drawn for basic biochemical measurements, including serum vitamin B_12_. The serum and plasma were separated within one hour by centrifugation at room temperature. The samples were then aliquoted into storage tubes and stored at a minimum of −20°C. The samples were later transported frozen to the National Institute for Health and Welfare and stored at −70°C until analyzed at the laboratory of the Disease Risk Unit.

Serum vitamin B_12_ was measured with an Architect ci82000 analyzer (Abbott Laboratories, Abbott Park, IL) using the Chemiluminescent Microparticle Immuno Assay (CMIA). The reference range was 138–652 pmol/L for the normal serum vitamin B_12_ level. The interassay coefficients of variation (CV) of B_12_ vitamin were 6.2% and 5.0% at the levels of 150 pmol/L and 380 pmol/L, respectively.

#### Other measurements

Height was measured to the nearest 0.1 cm, and weight was measured in light clothing to the nearest 0.1 kg. The body mass index (BMI) was calculated as weight (kg) divided by the square of height (m). Waist circumference was measured midway between the lowest rib margin and the iliac crest. Education was assessed according to years of education. The participants reported their marital status, and were categorized as married, single, separated or widowed. Employment status was inquired, and the number of employed participants was counted. Current smoking and alcohol consumption were assessed with self-administered questionnaires, and dichotomized (no or yes). Leisure- time physical activity (LTPA) was assessed with the question: “How much physical activity do you practice during leisure- time?” Response categories were: In my leisure- time I 1) read, watch television and do things that do not require physical activity; 2) walk, ride a bicycle or exercise in other ways requiring physical activity for at least four hours a week; 3) have physical activities to maintain my condition such as jogging, cross-country skiing, aerobics, swimming or ball games at least three hours a week; and 4) practice regularly for competitions in running, cross-country skiing, orienteering, ball games, or other heavy physical exercise several times a week. The intensity of LTPA was classified as low (category 1), moderate (category 2) or high (categories 3 and 4) [31]. A chronic diseases sum index was based on the question “Have you had any of the following diseases that have been diagnosed or treated by a doctor in the last 12 months?” The diseases included elevated blood pressure, heart failure, angina pectoris/other cardiovascular event, diabetes, cancer, bronchial asthma/emphysema and rheumatoid arthritis/other arthropathy/spinal diseases. The chronic diseases sum index ranged from 0 to 7 [32]. The use of antidepressive medications was also recorded.

#### Statistical analysis

The data are presented as means with standard deviations or counts with percentages. Groups were statistically compared using the *t*-test, permutation test or chi-squared test, as appropriate. The statistical significance between groups was B12 level evaluated by bootstrap type analysis of covariance (ANCOVA) with appropriate contrast Multinomial logistic regression was used to analyze the relative risk ratios (RRR) and their 95% confidence intervals (95% CI) for the presence of non-melancholic and melancholic DS with appropriate contrasts. Adjusted continuous relationship between vitamin B12 levels and BDI score was analyzed using regression analysis (with bootstrap based standard error), squared term of the BDI score was added to an equation.

The multinomial (polytomous) logistic regression model is an extension of the binomial logistic regression model and is used when the dependent variable has more than two nominal (unordered) categories.

## Results

The study population (N = 2806) included 429 subjects (15%) with a BDI score ≥10. Table 1 presents the sociodemographic characteristics of the two subgroups with BDI scores <10 or ≥10. Participants with elevated DS were more likely to be female or older, and to have a higher BMI, be less educated, unmarried or unemployed. They also used less alcohol and were less physically active than their non-depressed counterparts. Subjects with increased DS had a higher chronic diseases sum index, and more often used antidepressive medication.

**Table 1:** Demographic and clinical data at baseline according to depressive symptom status

	**Depressive symptoms status**		**P-value**
	**BDI score <10** **N = 2377**	**BDI score ≥10** **N = 429**	
Female, n (%)	1215 (51)	263 (61)	<0.001
Age, years, mean (SD)	59 (8)	61 (9)	<0.001
Body mass index (kg/m^2^), mean (SD)	27.3 (4.7)	28.6 (5.6)	<0.001
Education years, mean (SD)	11 (8.14)	10 (8.13)	<0.001
Marital status, n (%)			<0.001
Married	1842 (78)	286 (67)	
Single	181 (8)	47 (11)	
Separated	215 (9)	61 (14)	
Widowed	130 (5)	33 (8)	
Employed, n (%)	1229 (52)	113 (26)	<0.001
Current smoker, n (%)	508 (21)	111 (26)	0.038
Using alcohol, n (%)	1465 (62)	225 (52)	<0.001
Leisure time physical activity, n (%)			<0.001
Low	375 (16)	141 (35)	
Moderate	1391 (60)	218 (54)	
High	561 (24)	46 (11)	
Chronic diseases sum index (0–7), mean (SD)	0.60 (1.06)	1.12 (1.30)	<0.001
Use of antidepressive medication, n (%)	71 (3)	77 (18)	<0.001

Abbreviations. BDI-score ≥10 includes 291 subjects with non-melancholic DS and 138 subjects with melancholic DS.

BDI = Beck Depression Inventory.

DS = depressive symptoms.

**Figure 1:**
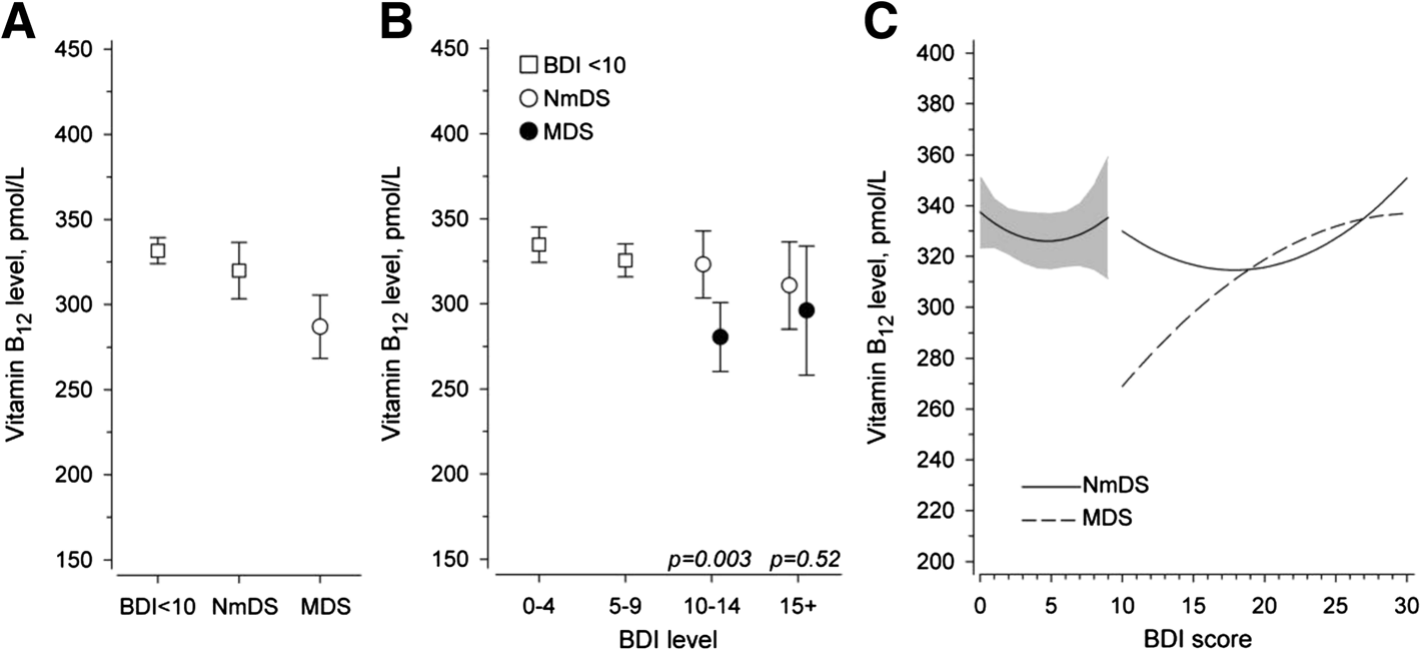
**The association of vitamin B**_**12 **_**levels with depressive and non-depressive symptoms, and with the severity of depressive symptoms based to BDI scores. A**: The association of vitamin B_12_ levels with the non-melancholic or the melancholic depressive symptoms and the non-depressive symptoms. **B**: The association of vitamin B_12_ levels with severity of the non-melancholic and the melancholic depressive symptoms according to the BDI-scores. **C**: Non-linear quadric model of the vitamin B12 levels versus BDI score. Model adjusted using age, sex, use of antidepressive medication and chronic diseases. The gray band gives the 95% confidence intervals. BDI, Beck Depression Inventory; NmDS, non-melancholic depressive symptoms; MDS, melancholic depressive symptoms.

Vitamin B_12_ levels differed between males and females (serum vitamin B_12_ 343 ± 168 pmol/L for females and 312 ± 171 pmol/L for males; p < 0.001). In order to further examine the vitamin B_12_ levels in subjects with depressive symptoms they were subdivided into groups with predominantly melancholic (N = 138) and non-melancholic DS (N = 291). The mean vitamin B_12_ level was 331±176 pmol/L in those without DS while the subjects with non-melancholic DS had a mean vitamin B_12_ level of 324 ± 135 pmol/L, and those with melancholic DS had the lowest mean vitamin B_12_ level of 292±112 pmol/L (p < 0.001 after adjusted for age, sex, use of antidepressive medication and chronic diseases sum index) (Figure 1A). The adjusted difference of vitamin B_12_ levels between the non-melancholic and the melancholic group was 33 pmol/L (95%CI 8 to 57, p = 0.008). No difference was found between those without DS and the non-melancholic group (Figure 1A). The vitamin B_12_ levels were significantly lower in the sub-group of melancholic depressive symptoms scoring 10–14 in the BDI. The association of vitamin B_12_ levels with the non-melancholic and melancholic depressive symptoms when subdivided according to the BDI-scores is presented in Figure 1B.

Figure 1C shows adjusted continuous relationship between vitamin B12 levels and BDI score.

In the multinomial regression analysis there was an independent linearly inverse association between the vitamin B_12_ tertiles and melancholic depressive symptoms: the relative risk ratio (RRR) was 2.75 (95% CI 1.66 to 4.56, p for linearity <0.001) in the lowest vitamin B_12_ level tertile as compared to the highest when those without DS formed the reference group (Table 2). There was no association between B_12_ vitamin tertiles and non-melancholic DS, since the RRR for the lowest vitamin B_12_ level tertile versus the highest was 1.20 (95% CI 0.86 to 1.66, p for linearity 0.28) (Table 2).

**Table 2:** Relative risk ratios and their 95% confidence intervals (95% CI) from multinomial regression analysis for having non-melancholic or melancholic depressive symptoms

**Variables**	**NmDS versus BDI < 10 RRR (95% CI)**	**P-value**	**MDS versus BDI < 10 RRR (95% CI)**	**P-value**
Vitamin B_12_ tertiles*				
I	1 (reference)	0.28#	1 (reference)	<0.001#
II	1.01 (0.72 to 1.41)		1.90 (1.21 to 3.22)	
III	1.20 (0.86 to 1.66)		2.75 (1.66 to 4.56)	
Male sex	0.59 (0.44 to 0.79)	<0.001	0.82 (0.54 to 1.23)	0.34
Age	1.02 (1.00 to 1.04)	0.041	1.02 (1.00 to 1.05)	0.091
BMI	1.02 (1.00 to 1.05)	0.11	0.98 (0.94 to 1.02)	0.32
Smoking	1.23 (0.88 to 1.71)	0.23	1.41 (0.91 to 2.20)	0.13
Using alcohol	1.09 (0.81 to 1.45)	0.59	0.84 (0.56 to 1.26)	0.39
LTPA		<0.001#		0.006#
I	1 (reference)		1 (reference)	
II	0.50 (0.37 to 0.68)		0.42 (0.27 to 0.64)	
III	0.24 (0.15 to 0.39)		0.43 (0.24 to 0.76)	
Years of education	0.98 (0.94 to 1.02)	0.24	1.00 (0.95 to 1.06)	0.99
Living alone	1.29 (0.95 to 1.75)	0.10	1.93 (1.30 to 2.88)	0.001
Energy intake	1.00 (1.00 to 1.00)	0.90	1.00 (1.00 to 1.00)	0.74
Use of antidepressive medication	5.51 (3.55 to 8.54)	<0.001	9.45 (5.63 to 15.86)	<0.001
Chronic diseases sum index	1.41 (1.26 to 1.59)	<0.001	1.21 (1.02 to 1.45)	0.031

* Gender specific tertiles: Male I > 340 pmol/L, II = 255–340 pmol/L, III < 255 pmol/L;

Female I > 380 pmol/L, II = 280–379 pmol/L, III = <280 pmol/L.

# P for linearity.

RRR = relative risk ratio.

BDI = Beck Depression Inventory.

NmDS = non-melancholic depressive symptoms.

MDS = melancholic depressive symptoms.

BMI = body mass index.

LTPA = leisure-time physical activity.

## Discussion

The novel finding in our population-based study, controlled for multiple potential confounders, was that vitamin B_12_ levels showed an independent linearly inverse association with the risk of melancholic DS but not with non-melancholic DS. This result is in line with the monoamine hypothesis of depressive disorders connecting a low vitamin B_12_ level with diminished synthesis of serotonin and other monoamines [4]. In our study we observed an approximately three-fold increased RRR for melancholic DS in the lowest vitamin B_12_ tertile. Thus, the risk is very much in the same range as the results from a recent study in which vitamin B_12_ deficiency appeared to be associated with the occurrence of DS (OR = 2.68) [18]. Two earlier studies reported similar, but somewhat lower risk levels (OR 2.05 and 1.64, respectively) [15,16]. Previously we have reported an association between low folate intake and increasing risk of melancholic DS in this material [36]. Homocysteine was not determined in this study.

All the previous studies showing positive relationships between vitamin B_12_ levels and DS or depressive disorders have been conducted among older populations [15-18]. On the other hand, most previous population-based studies not showing an association between vitamin B_12_ levels and depressive disorders or DS have been conducted on younger populations than that in our study [20,21,23]. Exceptions are an American and an Australian study that failed to detect this association among older populations [19,22]. These partly inconsistent results may suggest that the age of the study population is important although methodological differences in subject selection and in the measurement methods of depressive symptoms or depression, or in the B_12_ status, may also contribute. The distribution of depressive subtypes may also be important as in our study vitamin B_12_ levels were lower in the melancholic sub-group. In addition, the severity of DS is also relevant as the difference was significant only in the sub-group of mild to moderate depression i.e. 10–14 points in the BDI. The cut-off points for BDI tertiles were selected according to previous studies suggesting BDI-score of 10 [25,26] or 14 as proper cut of values [38,39]. Statistical reasons, such as a low number of subjects, or a large variety of vitamin B_12_ levels in the sub-group having BDI-scores ≥15 may have affected the significance of the result.

These previous results suggest that the elderly may be more vulnerable to low vitamin B_12_ levels. One plausible explanation for these findings is that vitamin B_12_ deficiency is more common in the aged. Its prevalence was 12% in the Finnish population (aged 65–100 years) compared to the finding that 5% of Canadians (age 6–79 years) were vitamin B_12_ deficient [40,41]. Lifestyle factors such as smoking, alcohol consumption and a vegetarian diet have been linked with anincreased risk of vitamin B_12_ deficiency in younger adults, [42,43] but no such association was recorded in an aged Finnish population [40]. No specific risk group for lower vitamin B_12_ levels could be defined among the aged [40]. It may also be possible that the brain effects of low vitamin B_12_ do not manifest until much later in life.

The strengths of our study include a large population-based sample containing middle-aged and elderly subjects with a substantial prevalence of DS. The study population was also geographically representative, covering both urban and rural districts in three study areas. In addition, the study data were comprehensively examined, and we used a WHO-based study methodology [37]. The BDI with a cut-off score of 10 points has also been shown to be a useful instrument for detecting DS in various adult populations [27-32].

The available evidence suggest some clinical utility and some validity for the concept of melancholic features [44]. Depression with melancholic or non-melancholic features can be established by using diagnostic interviews [45,46]. Instead,we used a summary score of melancholic symptoms in the BDI based on the DSM-IV- defined criteria for melancholic depression in order to examine the effect of the subtype of DS [33]. The way of dividing the DS into melancholic or non-melancholic DS has been applied in several previous studies as well [29,34-36].

Unfortunately, we were not able to present sensitivity analysis due to a dicotomous, and not a continuous method established in dividing DS into melancholic and non-melancholic DS. Factor analysis was not used because symtom composition of the resultant factors may be dependant of the types of samples being studied [33]. Furthermore, due to population-based design of the study the amount of subjects having lower scores in the BDI was high.

However, the criteria that guided the choice for melancholic DS need to be discussed more thoroughly. The chosen melancholic symptoms in the BDI are based on the DSM-IV- defined criteria for melancholic depression [47]. Although e.g. irritability can occur in the non-demented and demented older populations it may be quite near to agitation which is one the criteria for melancholic depression in DSM-IV [47,48]. The fatique and somatic factor symptoms, including irritability, may be major features of major and in particular of melancholic depression [49]. Irritability may be a symptom of mixed depression as well [50]. In addition, 11-21% of persons in Finland have DS assessed according to the BDI with the same, a rather low cut-off score of 10 points, which is in line with the prevalence of 15% shown in the present study [29,51].

In addition, as the study population was in advanced middle age, the generalizability of the results to younger age groups may be limited. Furthermore, due to the cross-sectional study design, we cannot make inferences of causality. However, cross-sectional studies can produce new associations or hypotheses that can be futher studied in observational settings.

## Conclusions

In our study we observed that a higher risk of melancholic depressive symptoms was associated with lower vitamin B_12_ levels. Our findings suggest that vitamin B_12_ may contribute to the pathogenesis of DS, although further studies are needed to evaluate the possible associations between DS and vitamin B_12_ levels among populations with different ages and depressive subtypes.

## Abbreviations

ANCOVA: Analysis of co-variance; BDI: the Beck depression inventory; BMI: Body mass index; CMIA: Chemiluminescent microparticle immuno assay; CV: Coefficients of variation; DS: Depressive symptoms; FIN-D2D: the Finnish diabetes prevention programme; LTPA: Leisure time physical activity; MDS: Melancholic depressive symptoms; MONICA: Monitoring trends and determinants in cardiovascular disease; NmDS: Non-melancholic depressive symptoms; RRR: Relative risk ratio; SAM: S-adenosylmethionine; WHO: World health organisation.

## Competing interests

The authors declare that they have no competing interests.

## Authors’ contributions

Authors HK, PM, HO and MV designed the study. Authors OK, YO and MV participated in recruiting and interviewing the patients. Authors JGE, OK, HK, JL, SM, PM, YO, MV, MV and JS wrote the article. Author HK undertook the statistical analysis. All authors contributed to and have approved the final manuscript.

## Pre-publication history

The pre-publication history for this paper can be accessed here:

http://www.biomedcentral.com/1471-244X/13/145/prepub
